# Prevention and management of postoperative delirium in adult patients: a best practice implementation project

**DOI:** 10.1590/1980-220X-REEUSP-2023-0426en

**Published:** 2025-02-24

**Authors:** Juliana Rizzo Gnatta, Sarha de Oliveira Gonçalves Paes, Maria Fernanda de Oliveira Faria, Domingos Dias Cicarelli, Renata Veloso Silva Laurino, Lina Hamano, Vilanice Alves de Araújo Püschel, Vanessa de Brito Poveda

**Affiliations:** 1 Universidade de São Paulo, Escola de Enfermagem, Departamento de Enfermagem Médico-Cirúrgica, São Paulo, SP, Brazil.; 2 Universidade de São Paulo, Escola de Enfermagem, São Paulo, SP, Brazil.; 3 Universidade de São Paulo, Hospital Universitário, São Paulo, SP, Brazil.; 4 Centro Brasileiro para o Cuidado à Saúde Baseado em Evidências: Centro de Excelência do JBI, JBI Brasil, São Paulo, SP, Brazil.

**Keywords:** Delirium, Postanesthesia Nursing, Postoperative Cognitive Complications, Anesthesia Recovery Period, Recovery Room, Interdisciplinary Research, Delírio, Enfermería Posanestésica, Complicaciones Cognitivas Postoperatorias, Periodo de Recuperación de la Anestesia, Sala de Recuperación, Investigación Interdisciplinaria

## Abstract

**Objective:**

To describe the process of implementing evidence-based best practices to improve the prevention and management of Postoperative Delirium in the Post-Anesthesia Care Unit.

**Method:**

Report on an evidence implementation project applying the JBI based on the audit and feedback process, with a structured approach to identifying and managing barriers in accordance with recommended clinical practices. Medical records, electronic nursing record systems, and staff interviews were used to assess compliance rates.

**Results:**

In the baseline audit, a zero compliance rate was found in most criteria in relation to the best evidence. Following this phase, interprofessional training was carried out, a validated tool was provided to identify patients at risk of delirium, and changes were made to the electronic nursing records system. In the first follow-up audit, there was an increase in the compliance rate in four of the nine criteria audited. Four criteria achieved 100% compliance in the second follow-up audit.

**Conclusion:**

Best practices were implemented that contributed to improving the prevention and management of Postoperative Delirium. Subsequent to new training and re-auditing after one year, an increase in adherence to best practices was observed.

## RESUMEN

**Objetivo:**

Describir el proceso de implementación de mejores prácticas basadas en evidencia para mejorar la prevención y el manejo del delirio posoperatorio en la sala de recuperación posanestésica.

**Método:**

Informe de un proyecto de implementación de evidencia aplicando el JBI basado en el proceso de auditoría y retroalimentación, con un enfoque estructurado para identificar y gestionar barreras de acuerdo con las prácticas clínicas recomendadas. Se utilizaron registros médicos, sistemas electrónicos de registros de enfermería y entrevistas al personal para evaluar las tasas de cumplimiento.

**Resultados:**

En la auditoría de referencia, se encontró una tasa de cumplimiento cero en la mayoría de los criterios en relación con la mejor evidencia. Luego de esta fase, se realizó capacitación interprofesional, provisión de una herramienta validada para identificar pacientes con riesgo de delirio y cambios en el sistema de registros electrónicos de enfermería. En la primera auditoría de seguimiento se produjo un aumento en la tasa de cumplimiento en cuatro de los nueve criterios auditados. Cuatro criterios lograron un cumplimiento del 100% en la segunda auditoría de seguimiento.

**Conclusión:**

Se implementaron mejores prácticas que contribuyeron a mejorar la prevención y el manejo del Delirio Postoperatorio. Después de una nueva capacitación y una nueva auditoría tras un año, se observó un aumento en el cumplimiento de las mejores prácticas.

## INTRODUCTION

Delirium is a neuroinflammatory clinical condition characterized by inattention and an unstable level of consciousness, presenting as a clinical picture subject to fluctuations throughout the day. It can be classified into two types: hyperactive and hypoactive. Hypoactive delirium is characterized by drowsiness, with lethargic and reduced movements. Hyperactive delirium is characterized by psychomotor agitation, visual hallucinations, and hypervigilance; both may manifest immediately upon awakening from anesthesia in the Post-Anesthesia Care Unit (PACU)^(1)^.

The incidence of delirium varies according to the type of surgery, mainly affecting older people undergoing major surgical procedures, such as cardiac, general, orthopedic and vascular surgeries, with involvement varying between 10% and 62%^(2,3,4,5)^.

There are factors that predispose patients to the development or increased risk of Post-Operative Delirium (POD). According to the clinical guidelines proposed by the National Institute for Health and Care Excellence (NICE) for delirium, the main risk factors for developing it are age over 65, chronic cognitive decline or dementia, current hip fracture, serious illness, and a deteriorating or at risk of deteriorating clinical condition^(6)^. Moreover, predisposing risk factors that increase the incidence of POD in the PACU include: longer duration of the intraoperative period, such as that which occurs in major or emergency surgeries, comorbidities, preoperative anemia, hypothermia, postoperative pain, and high fasting blood glucose levels^(7,8)^.

Understanding the causality of POD is, in fact, important to offer safe perioperative care to patients and prevent the consequences arising from it, such as: prolonged hospitalization, loss of independence, reduced cognitive function, and death^(8,9)^. Therefore, screening with early identification and treatment of POD helps prevent future complications. To this end, the application of validated instruments allows for agility and precision in identification, providing the timely beginning of treatment, while still in the PACU, requiring interprofessional work for its correct management^(7,10)^.

For the prevention of future complications^(7)^, the identification of individuals at increased risk for delirium allows early interventions, improving the patient’s clinical results and, consequently, the prognosis^(9)^.

Joanna Briggs Institute (JBI) evidence methodology consists of offering a theoretical approach focused on clinical evidence, using evidence and implementing it in practice, through clinical auditing and the use of tools to facilitate and guarantee records of changes. It is considered a process for improving the quality of patient care and the results obtained, in addition to reducing costs and increasing the satisfaction of those involved in the improvement process.

Based on systematic review and clinical guidelines^(7,10,11,12,13)^, the JBI, through the publication of an evidence summary^(14)^, makes recommendations classified into two levels: Grade A, which means a strong recommendation, and Grade B, which means a weak recommendation. JBI recommends using a validated cognitive screening test to identify at-risk patients, such as the *Confusion Assessment Method* (CAM-ICU) or the *Nursing Delirium Screening Scale* (Nu-DESC) for the detection of POD, and the test should be readily available in the PACU (GRADE B).

Regarding best clinical practices, the *Evidence Summary* produced by JBI^(14)^ recommends that the depth of anesthesia be minimized and monitored during surgery (GRADE A). Additionally, multimodal postoperative pain control and low-dose haloperidol or atypical neuroleptics are recommended when necessary for the treatment of POD (GRADE B). The recommendations described were based on evidence from the literature, from two systematic reviews^(10,11)^, two randomized clinical trials^(12,13)^, a retrospective observational study^(15)^, and an evidence-based clinical guideline^(7)^.

Identifying patients at risk of developing POD through screening and detection contributes to early intervention and management, improved critical patient care, reduced PACU length of stay, hospitalization and postoperative complications, and improved patient status at hospital discharge.

Anesthesiologists and nurses play an important role in POD prevention and management, assessing vital signs, hypoxia, hydration, nutrition (when indicated), rest and spatial-temporal orientation, especially in cases of emergency surgery^(16)^, thus intervening to provide the best possible care based on the best evidence, contributing to better management of the surgical patient in the immediate postoperative period, which is the focus of this article.

This evidence implementation project is interdisciplinary, as it presents multiple possibilities for implementation, whether from a theoretical or practical point of view, teamwork or the subject’s individual attitude.

The multiple conceptions of interdisciplinarity converge towards the idea of integrality, be it of knowledge, learning, individuals, or disciplinary areas. It is necessary to invest in knowledge about interdisciplinarity, either through individual, collective, institutional or governmental attitude, to implement best practices in health institutions^(17)^.

The objective of this study was to describe the process of implementing evidence-based best practices to improve the prevention and management of Postoperative Delirium in the Post-Anesthesia Care Unit of a university hospital.

## METHOD

### Design of Study

This is a report of an evidence implementation project that used the JBI evidence implementation methodology, which is based on the audit process and *feedback*, along with a structured approach to identifying and managing barriers in line with recommended clinical practices. It consists of seven steps: (1) identification of the practice area for change and of the problem with the stakeholders, (2) engagement of change agents, (3) assessment of context and readiness for change (i.e. situational analysis), (4) review of practice (i.e. baseline audit) against evidence-based audit criteria, (5) implementation of changes in practice applying the GRiP tool, (6) reassessment of practice through a follow-up audit, and (7) consideration of the sustainability of changes in practice.

In this model, a clinical audit is adopted that proposes a comparison between the compliance rates of current practice with recommended best practices, before (baseline audit) and after (follow-up audit) the implementation of the evidence, to assess adherence to best practices. Periodic re-audits are recommended to verify the sustainability of the implementation of the evidence over time. The process is conducted using JBI software tools for data collection and analysis, such as *Practical Application of Clinical Evidence System* (PACES)^(18)^, and to identify barriers to change and propose strategies for using evidence in practice, such as *Getting Research into Practice* (GRiP).

The audit structure and feedback based on the seven steps of the JBI model involved a three-phase process: (1) planning – identifying the practice area for change and the problem with the stakeholders, engagement of change agents and assessment of context and readiness for change; (2) baseline assessment and implementation – review of practice (baseline audit) and implementation of changes in practice applying the GRiP tool; and (3) impact and sustainability assessment – reassessment of practice through a follow-up audit and consideration of the sustainability of changes in practice.

### Local and Sample

This project was developed at the PACU of the University Hospital of the Universidade de São Paulo (HU-USP). The hospital is located in the metropolitan region of São Paulo, Brazil and, during the period evaluated, had 203 beds. The PACU has seven beds and is located in the Surgical Suite, where an average of 300 surgeries/month take place in the specialties of orthopedics, general surgery, otorhinolaryngology, gynecology, and ophthalmology. To compose the study sample, all professionals who worked in the unit during the period evaluated were included. The team was made up of 10 nurses, 39 nursing technicians, and 21 anesthesiologists, organized on a rotation basis. No exclusion criteria were established for participation.

The PACU nursing team on each shift consists of one nurse, one nursing technician per shift (morning, afternoon, and night), and one anesthesiologist. Nurses are responsible for implementing the nursing process (assessment, nursing diagnoses, planning, implementation and evaluation) and providing nursing care, and nursing technicians assist nurses in low to moderate complexity interventions, such as checking vital signs and basic nursing care. Anesthesiologists are responsible for the clinical evaluation and discharge of the patient from the PACU to the inpatient unit, depending on the level of criticality.

In 2015, the board of the Clinical Nursing Division developed a protocol for the assessment and management of delirium in clinical patients. Although the implementation of best practices in a surgical suite enhances the improvement in the institution’s quality of care, the implementation of the aforementioned protocol was not extended to all units. Currently, the protocol for medical patients continues to be applied, presenting good results in healthcare practice. This was one of the reasons for carrying out this other project at the institution. Medical or surgical patients benefit from a systematic and appropriate assessment and management of delirium, reducing adverse events and increasing safety, as well as contributing to the quality of the institution’s processes.

### Phase 1: Planning

The audit team involved stakeholder engagement and identification of relevant team members.

The project team consisted of:

– A teacher with experience in Perioperative Nursing, project leader, and who had previously worked as an assistant nurse in the institution’s PACU;
– A teacher with experience in Perioperative Nursing, who evaluated strategies to increase compliance with the recommended audit criteria;
– Three clinical managers, one head of the Surgical Nursing Division and two from the Anesthesiology Division who provided human and physical resources for the audits, developed the implementation project, and participated in team training;
– The Chief Nursing Officer of the Surgical Suite, who made the necessary changes to the electronic medical record system to support nursing care records related to POD screening and detection (CAM-ICU score) and oversaw the implementation project;
– A nursing resident, who was responsible for supporting educational training, monitoring difficulties, doubts and barriers during screening and POD assessment.

The project leader, the nursing resident and the two anesthesiologists were responsible for designing the project, preparing and conducting the training program on *delirium*, organization of data collection and preparation of the implementation report.

The evidence-based audit criteria were derived from the JBI evidence summary on best practice recommendations regarding screening and detection of *delirium* in the PACU^(14)^. To define the current level of adherence to best practices, all nine audit criteria included in JBI-PACES were considered, as shown in Chart 1 ^(14)^.

**Chart 1:** Audit criteria, sample, and method used to assess compliance with best practices for prevention and management of POD in adult patients in the PACU – Brazil, 2021.

Audit criteria*	Sample	Method used to measure percentage compliance with best practices
1. The depth of sedation during anesthesia is monitored subjectively or objectively.	• Baseline audit: 10 observations of anesthesiologists during the procedure with patients with risk factors for POD. • Follow-up audit: 10 observations of anesthesiologists during the procedure with patients with risk factors for POD.	• Direct observation by the anesthesiologist during the procedure in patients with risk factors for POD to verify whether the depth of sedation was monitored through subjective or objective measurement. Subjective measure: “Yes” – application of the sedation scale; “No” – no record. Objective measure: “Yes” – use of equipment to guide anesthesia (e.g.: *Bispectral Index Monitoring* – BIS); “No” − not used.
2. Preoperative risk assessment for POD is performed.	• Baseline audit: 10 nursing records of patients with risk factors for POD. • Baseline audit: 10 nursing records of patients with risk factors for POD.	• Review of nursing records to verify if there was a nursing diagnosis of “Risk for acute confusion”. “Yes” – presence of the nursing diagnosis “Risk for acute confusion”; “No” – absence of the nursing diagnosis “Risk for acute confusion”.
3. Screening for POD is performed as early as possible in the PACU.	• Baseline audit: number of patients with positive screening for POD in 10 records in electronic nursing systems of patients at risk of POD. • Follow-up audit: number of patients with positive screening for POD in 10 records in electronic nursing systems of patients at risk of POD.	• Review of electronic nursing record systems for patients at risk for POD. “Yes” – if there is a CAM-ICU score; “No” – if there is no CAM-ICU score.
4. The tools *Nursing Delirium Screening Scale* (Nu-DESC) or *Confusion Assessment Method* (CAM-ICU) are used for screening.	• Baseline audit: number of patients screened for POD using the CAM-ICU tool across 10 patient observations. • Follow-up audit: number of patients screened for POD using the CAM-ICU tool across 10 patient observations.	• Nursing documentation in the patient's medical record. “Yes” – presence of CAM-ICU score recorded in the electronic nursing record system; “No” – absence of CAM-ICU score recorded in the electronic nursing record system.
5. The screening tool is easily accessible in the PACU.	• Baseline audit: presence of printed CAM-ICU tool available in the PACU on 10 occasions. • Follow-up audit: presence of printed CAM-ICU tool available in the PACU on 10 occasions.	• Presence of copies of the CAM-ICU tool in the PACU. “Yes” – Copy of the CAM-ICU tool available in the PACU to assess patients with risk factors for POD; “No” – Copy of the CAM-ICU tool not available in the PACU to assess patients with risk factors for POD.
6. Clinicians receive training in the use of Nu-DESC and/or CAM-ICU.	• Baseline audit: 10 nurses. • Follow-up audit: 10 nurses.	• Interview with 10 nurses using a structured form to verify whether the professional received training for the use of CAM-ICU in the last year. “Yes” – if the professional received training; “No” – if the professional did not receive training.
7. Physicians receive training on DSM-5 and/or ICD-10 reference standards.	• Baseline audit: 10 anesthesiologists. • Follow-up audit: 10 anesthesiologists.	• Interview with 10 anesthesiologists using a structured form. “Yes” – if the professional received training on DSM-5 and/or ICD-10 in the past year. “No” – if the professional did not receive training on DSM-5 and/or ICD-10 in the past year.
8. Multimodal postoperative pain control is administered.	• Baseline audit: 10 post-operative medical prescriptions accompanied by the respective anesthetic prescription. • Follow-up audit: 10 post-operative medical prescriptions accompanied by the respective anesthetic prescription.	• Review of 10 postoperative medical prescriptions for patients at risk of POD who reported pain during their PACU stay. “Yes” – if the patient received an opioid analgesic combined with a non-opioid; “No” – if the patient did not receive an opioid analgesic combined with a non-opioid.
9. When necessary, low-dose haloperidol or low-dose atypical neuroleptics are given to treat POD.	• Baseline audit: 10 postoperative medical prescriptions of patients with POD who received haloperidol or atypical neuroleptics in the PACU. • Follow-up audit: 10 postoperative medical prescriptions of patients with POD who received haloperidol or atypical neuroleptics in the PACU.	• Review of 10 postoperative medical prescriptions of patients with POD who received haloperidol or atypical neuroleptics in the PACU. “Yes” – if the patient received haloperidol or low-dose atypical neuroleptics; “No” – if the patient did not receive haloperidol or low-dose atypical neuroleptics.

*Audit criteria: based on JBI evidence summary of best practice recommendations^(14)^.

Source: the authors.

The baseline audit included medical records of 10 patients, aged 18 years or older, who had the nursing diagnosis “Risk for acute confusion” and who presented at least one risk factor for POD identified by the nursing resident at the time of the patient’s admission and validated by the project’s lead nurse. Ten professionals from each category (nurses and anesthesiologists) working in the PACU and who treated these patients were also included.

### Data Collection

A printed instrument created by the researchers was used to collect data (Chart 2) and the data were entered into PACES. Adherence was observed by the project leader and the nursing resident and data collection took place in November 2021 using the medical records of patients who presented at least one risk factor for POD: age greater than or equal to 65 years, cognitive impairment or diagnosis of dementia, current hip fracture, and presence of severe disease^(6)^. The presence of the information was recorded as “Yes”, to later measure the percentage of adherence to best practices.

**Chart 2:** Data collection instrument to assess compliance with best practices for prevention and management of POD in adult patients in the PACU – Brazil, 2021.

Criteria – Patient Records	Yes	No
1. Is there a record in the Perioperative Nursing Process (SAEP) of the Nursing diagnosis “Risk for acute confusion” in the field for patients at risk of postoperative delirium? (Preoperative risk assessment for delirium – criterion 2)		
2. Is there a record in the electronic (PROCEnf) or physical medical record of the application of an instrument for early screening of delirium (Ex: Nu-DESC or CAM) in PACU? (criterion 3)		
3. Is there a record in the electronic (PROCEnf) or physical medical record of the application of the Nu-DESC or CAM tools during the patient's stay in the PACU? (criterion 4)		
4. Is there a record in the anesthesia record or in the postoperative medical prescription of the administration of a non-opioid analgesic in combination with an opioid? (criterion 8)		
5. Is there a record in the medical prescription of post-operative haloperidol or low-dose atypical neuroleptics? (criterion 9)		
**Criteria – Direct Observation**		
6. Did the anesthesiologist monitor the depth of anesthesia using subjective (e.g., application of a validated scale for measuring sedation) or objective (e.g., BIS) measurements? (criterion 1)		
7. Are printed copies of the Nu-DESC or CAM tools readily accessible or in the electronic patient data recording system (PROCEnf)? (criterion 5)		
**Criterium – Questioning the Anesthesiologist**		
8. In the past 24 months, have you received any training on the DSM-5 and/or ICD-10 reference standards? (criterion 7)		
**Criteria – Questioning the Nursing Professional**		
9. In the last 24 months, have you completed any training on the application of CAM or Nu-DESC tools for the detection of delirium? (criterion 6)		

### Phase 2: Baseline Assessment and Implementation

The results of the baseline audit were analyzed and shared with the implementation team to identify barriers to implementing best practices.

The JBI GRiP tool was applied to document the barriers encountered, the strategies implemented and the resources needed to overcome them, with the aim of improving compliance with the audited criteria. The barriers were identified by the group of professionals who worked at the study site.

Approximately thirty days after training completion, a new audit was conducted using the same criteria as the baseline audit.

### Phase 3: Impact and Sustainability Assessment

The first follow-up audit was carried out in November 2021. The same evidence-based audit criteria, sampling structure, and data collection personnel (project leader and nursing resident) used in the baseline audit were applied to assess adherence to best practices in the follow-up audits. After the follow-up audit was completed, the results were compared with the baseline audit to assess changes.

The second follow-up audit took place in November 2022. Before this audit, new training was provided to the entire team, as new professionals joined the PACU after the first follow-up audit, because some professionals who had already been trained left the unit. Professionals were trained at appropriate times during their shifts, so as not to interfere with work activities. Training was carried out by the project’s resident nurse and, subsequently, data were collected based on the same evidence-based audit criteria, sampling structure and baseline audit to assess adherence to best practices. After completion of the second follow-up audit, the results were analyzed and compared with the baseline audit.

### Data Analysis

Data were entered into JBI-PACES and a report containing the percentage of compliance for each baseline audit criterion was compared with the percentage from follow-up audits to assess the change in clinical practice regarding the prevention and management of POD.

### Ethical Aspects

This project was submitted and approved by the Research Ethics Committee of HU/USP on 05/21/2021 (registration number 4.729.079), guaranteeing data confidentiality, as well as the non-identification of participants.

After authorization from the institutions selected for this study, based on Resolution No. 466 of December 12, 2012, all patients and health professionals were informed about the purpose of the research, expressing their agreement to participate in the investigation, by signing the free and informed consent form (FICF), in two copies, one of which was given to the participant and the other remained in the possession of the researcher.

## RESULTS

### Phase 1: Planning

The results of the baseline audit were analyzed using the percentage of compliance for each audit criterion prior to the interprofessional training program. Each audit criterion relating to screening, evaluation and management of the delirium was prospectively evaluated in 10 consecutive medical records of patients admitted to the PACU who presented at least one risk factor for POD or with 10 professionals from each category who cared for the patient.

None of the medical records evaluated identified compliance with audit criteria 1 to 7. The zero compliance rate in most of the audit criteria (in 7 of the 9 criteria – 77.8%) of the best practice was expected, since the POD was not being assessed by any valid instrument before the development of the implementation project.

Regarding the training of staff (nurses and anesthesiologists) on how to use the recommended tool to assess delirium, none of the 10 nurses had been previously trained to apply the CAM-ICU and none of the anesthesiologists had been trained in the DSM-5 and/or ICD-10 reference standards.

### Phase 2: Baseline Assessment and Implementation

Chart 3 presents the four barriers faced in implementing the evidence, accompanied by strategies, resources, and results achieved.

**Chart 3:** Strategies for putting research into practice – *Getting Research into Practice* (GRiP) – Brazil, 2021.

Barriers	Strategy	Resources	Results
1. Most healthcare professionals were unaware of the risk factors for POD and how to apply and interpret the CAM-ICU tool.	• Development of formal interprofessional training and monitoring of professionals at the point of care to emphasize the application and interpretation of the instrument. • Development of an algorithm to screen and assess the risk of POD.	• Formal one-hour education session involving a PowerPoint presentation, an educational video, showing the practical application of CAM-ICU. • Application of the CAM-ICU algorithm and tool, *on site*, in a patient in the PACU.	• Health professionals were aware of the risk factors for delirium. This outcome was measured by verifying whether patients who presented at least one risk factor for POD had the nursing diagnoses “Risk for acute confusion” or “Acute confusion” recorded in their medical records.
2. CAM-ICU was not available in the PACU.	• Provision of printed copies of the CAM-ICU in the PACU.	• Laminated copies of the CAM-ICU tool were made available in the PACU.	• Printed CAM-ICU was available at the unit. This result was measured by checking whether the copy was available on the drive.
3. Screening and evaluation of delirium were not recorded in the medical records.	• Changes to the electronic nursing record system to show CAM-ICU scores linked to appropriate nursing diagnoses related to delirium (“Risk of acute confusion” or “Acute confusion”).	• Support from the head nurse. • IT support.	• The number of nursing diagnoses for “Risk for acute confusion” or “Acute confusion” identified and cases of POD increased significantly among adult patients in the PACU. This outcome was measured by checking patient medical records and electronic nursing records.
4. There is no equipment available at the institution to monitor the depth of sedation (BIS*).	• Arrange for the purchase of equipment to monitor the depth of sedation (BIS*).	• Support from the head nurse. • Support from clinical lead anesthesiologists.	• Acquisition of equipment (BIS*). This outcome was measured by verifying whether patients who presented at least one risk factor for POD were monitored by BIS during the anesthetic-surgical procedure.

*BIS = Bispectral Index Monitoring.

The main barrier identified by the group was the professionals’ lack of knowledge about the risk factors for delirium, assessment by CAM-ICU, and management.

According to the initial audit, no nurse or anesthesiologist had previously been trained in screening and assessment of delirium based on recommended risk criteria and the CAM-ICU tool. For this reason, interprofessional training was carried out by the project leader, the nursing resident and the two anesthesiologist members who made up the project team, supported by the other members of the implementation team. The educational strategy involved a one-hour formal education session on risk factors, screening, assessment and management of POD, a video presentation showing practical application of the tool and an on site demonstration in a patient with “Risk of acute confusion” admitted to the PACU, using an algorithm developed for the project by the researcher and applying the CAM-ICU tool.

All nurses on each work shift (morning, afternoon, and night) were trained. In the case of anesthesiologists, only 85.7% completed the training because three professionals were on vacation or away from their work activities during the implementation process.

Another relevant barrier was the nurses’ lack of knowledge about how to apply and interpret the CAM-ICU. Furthermore, the CAM-ICU tool was not available in the PACU. To overcome this barrier, the printed CAM-ICU was made available to the unit, training was provided to the team, and an algorithm was developed to guide the professional to screen, evaluate and classify the patient if he was at risk or if he presented delirium, and, in this case, adopt non-pharmacological measures and communicate with the anesthesiologist, so that he can indicate the pharmacological measures.

To overcome the barrier to screening and assessment of delirium regarding the medical record, changes were made to the electronic nursing process record system. The head nurse and the IT service were responsible for supporting this work.

After applying the CAM-ICU tool, nurses were responsible for identifying the nursing diagnoses “Risk for acute confusion” (NANDA taxonomy, code 00173) or “Acute confusion” (NANDA taxonomy, code 00128)^(19)^ and registering in the printed medical record. CAM-ICU negative patients were diagnosed as presenting “Risk for acute confusion” and CAM-ICU positive patients were diagnosed as having “Acute confusion”. The nurse then filled in the CAM-ICU score in the electronic nursing process record system.

At the hospital, there was no equipment available to monitor the depth of sedation. As the acquisition of this type of equipment would contribute to improving care practices and bring greater safety to the patient, the acquisition of the equipment *Bispectral Index Monitoring* (BIS) was requested.

Thus, 10 nurses (100.0%) and 21 anesthesiologists were trained (85.7%) on identification of risk factors, practical application of the CAM-ICU tool, non-pharmacological and pharmacological approaches. Those who were on vacation did not participate in the educational intervention (three professionals).

### Phase 3: Impact and Sustainability Assessment

**Figure 1:**
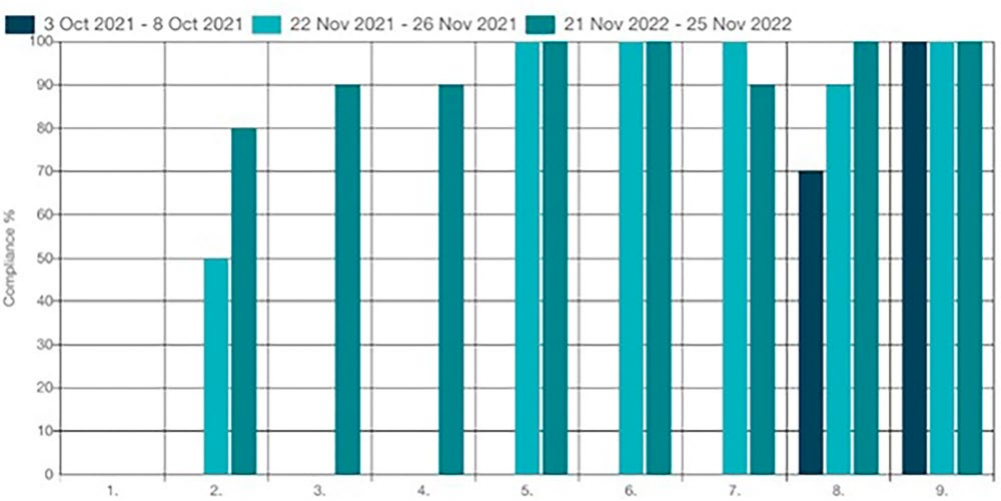
Compliance (%) of the baseline audit, first and second follow-up audits with the best practice criteria for prevention and management of POD in adult patients in the PACU – Brazil, 2022.

The results of the follow-up audit were analyzed using the percentage of compliance for the nine audit criteria. Each audit criterion was assessed in 10 records of consecutive patients admitted to the PACU with at least one risk factor for POD or through interview with 10 nurses and 10 anesthesiologists. Figure 1 shows the compliance rates of the second follow-up audit (re-audit) compared to the baseline audit and the first follow-up audit.

Audited criteria (legend):

1- The depth of sedation during anesthesia is monitored subjectively or objectively.

2- Preoperative risk assessment for POD is performed.

3- Screening for POD is performed as early as possible in the PACU.

4- The tools Nursing Delirium Screening Scale (Nu-DESC) or the Confusion Assessment Method (CAM-ICU) are used for screening.

5- The screening tool is easily accessible in the PACU.

6- Clinicians receive training in the use of Nu-DESC and/or CAM-ICU.

7- Physicians receive training on DSM-5 and/or ICD-10 reference standards

8- Multimodal postoperative pain control is administered.

9- When necessary, low-dose haloperidol or low-dose atypical neuroleptics are administered to treat POD.

In relation to the baseline audit, there was an improvement in compliance in four of the nine audited criteria.

As the project was carried out during the COVID-19 pandemic, when resources were scarce, and in a public institution, where the purchasing process is carried out through a bidding process involving long deadlines, the acquisition of equipment during the project period was hampered. Therefore, adherence to criterion 1 in the follow-up audit was zero because the BIS application and acquisition process is still ongoing.

There was an increase in seven of the nine best practice criteria for prevention and management of POD in the PACU. Adherence to criterion 1 in the second follow-up audit was also zero because BIS was not purchased to improve clinical practice.

## Discussion

An evaluation of an interprofessional training program to improve practices in POD prevention and management in adult surgical patients in the PACU was carried out, analyzing the criteria for its implementation. In the baseline audit, non-compliance was revealed with the vast majority of the audited criteria according to the best available evidence, demonstrating that the entire sample of patients undergoing surgical procedures was not systematically screened for potential risk factors for the development of POD.

Although knowledge about the mechanisms underlying the pathophysiology of delirium is limited, including abnormal metabolic events such as imbalance and neuroinflammatory disorders^(19)^, the negative repercussions on the clinical condition of surgical patients become irrefutable. Among these repercussions, the following stand out: higher morbidity and mortality rates, prolonged hospital stays, progressive cognitive decline and increased hospitalization costs, which result in significant negative implications for the individual’s rehabilitation and prognosis^(19)^.

As the risk predictors for the development of POD increase, associated with greater surgical complexity, it becomes essential to implement well-established screening protocols and processes involving the interprofessional team working in the perioperative period^(20)^. Continuing and permanent education of professionals as a facilitating strategy undertaken by health services is essential for detecting patients at high risk of developing POD in the PACU, thus contributing to limiting its incidence and offering excellent care^(20,21,22)^.

Studies corroborate, above all, the relevance of nurses in the screening and perioperative evaluation of groups with the greatest potential risk for developing delirium, since these professionals are responsible for the largest amount of time dedicated to providing and systematizing direct care to patients^(23,24,25)^. Findings in a similar study support that baseline and follow-up audits, combined with a training program in delirium and changes in electronic nursing records, increased adherence rates related to evidence-based practice for screening at-risk patients and assessing delirium^(24)^.

Complementing nurses’ work, members of the anesthesiology team are responsible for guiding patients and family members about potential risk factors that may directly affect discharge management and planning, due to the consequences of POD^(21)^.

The baseline audit showed that, although nurses play an important role in POD prevention, detection, and management^(23)^, there are gaps in the knowledge of these professionals on this topic. This is corroborated by the results found in the baseline audit, indicating that nurses, in their entirety, did not receive any type of training on delirium. Therefore, strategies were developed to provide and establish evidence-based practice as an indispensable precept in the work of these professionals, consecutively improving operational systems and, above all, the systematization of nursing care records.

Through the participation of professionals in training, it was possible to define POD prevention and management strategies, providing an opportunity to increase adherence to recommended good practices. Continuing health education is an agent that changes realities, while its implementation in institutional environments provides employees with resources, motivations, new practical and theoretical experiences and, consequently, empowerment of the team, creating an environment of mutual co-responsibility and collaboration^(25)^.

In view of the above, the training process for nurses and anesthesiologists was designed and executed by the implementation project team, who used different teaching strategies, such as: oral presentation to address the main topics on delirium applied to the surgical context, such as characterization, symptomatology, incidence, prevalence and associated outcomes; video explaining the applicability of the predictive tool of delirium − CAM-ICU − and algorithm for carrying out the screening, prevention and management steps of delirium. Professionals also participated in the on site application of the CAM-ICU tool and subsequent discussion based on the questions and doubts that arose during the practice. When applying CAM-ICU immediately after training, professionals took around seven to eight minutes to complete it. Following familiarization with the application of the tool, the time spent was around four to five minutes.

An African study highlights the importance of on site training in the health professionals’ clinical practice, demonstrating a significant increase in the effectiveness of training strategies to improve professionals’ practices^(22)^. This strategy is in line with the results obtained in this project, which observed an increase in the percentages of compliance with the audited criteria based on best practices after on-site training.

The continuous provision of a reliable tool applied in clinical practice^(7)^ to identify the delirium was essential to support the team in the systematic assessment for POD prevention and management, designed in the admission and post-anesthetic recovery environments. This strategy helped to facilitate the decision-making process by professionals during the patients’ post-anesthetic recovery period. Furthermore, it was possible to find that the training program developed favored POD screening and evaluation in the PACU in the surgical suite, since there was an increase in the percentages of compliance with best practices.

Regarding nursing care recording tools, it was important to update the tool intended for the Perioperative Nursing Process (SAEP), with the inclusion of the nursing diagnoses “Risk for acute confusion” and “Acute confusion” associated with the description of the predisposing factors for the development of delirium in the postoperative period. In addition, a field was included to describe the result of the CAM-ICU assessment in the institutional electronic nursing operating system, in the “Cognition” domain, thus contributing to documenting the assessment performed and facilitating communication between shifts and units, since the shift is configured by the principle of continuity^(25)^.

In line with a previous study on delirium, the results obtained in the second follow-up audit reached a compliance rate of over 80%^(26)^, which reflects the success of the training program carried out. However, although the level of compliance was high, it was not possible to achieve absolute rates, which may have occurred as a result of the situational context of a public organization immersed in a pandemic context.

As limitations, it is worth highlighting that, at the time of the project’s implementation, the world was experiencing one of the largest pandemics ever seen, characterized by hospital overcrowding. As a consequence, there was a contingency of human and material resources, which required reallocation and prioritization of expenses. Thus, requests for the acquisition of materials to improve clinical practice were put on the back burner, as was the case with BIS. One of the recommendations for best practices for preventing and managing POD (criterion 1) was based on the use of BIS so that the depth of anesthesia could be monitored, which would allow more reliable titration of the anesthetic drugs used and could reduce the occurrence of delirium^(20)^.

As a consequence of the pandemic, there was also a reduction in surgical flow. It is possible that, given such a high demand for assistance, the team prioritized some actions to the detriment of others in view of the scenario of multiple aggravating factors experienced, which may have influenced the lack of full adherence to the audit criteria, especially as it is a teaching hospital and linked to the best university in Latin America.

Furthermore, changing some team members and carrying out new training after a year may compromise the reliability of the results of this research. To minimize this risk, it would be necessary to adopt training provided annually to obtain better results in the institution evaluated. It should be noted that, as there was new training of the interprofessional team, it was not possible to verify the maintenance of the practices implemented during the one-year period. However, it can be seen that new training resulted in higher rates of compliance with best practices (re-audit) when compared with the data obtained in the first audit.

Given the results obtained in this study, it was evident that adherence to best practices is possible and feasible for identifying patients at high risk for developing POD in the PACU and that prevention and interprofessional management strategies should be implemented to prevent the occurrence of delirium in surgical patients.

## CONCLUSION

Best practices were implemented that contributed to improving the prevention and management of Postoperative Delirium in the Post-Anesthesia Care Unit. Subsequent to new training and re-auditing after one year, an increase in adherence to best practices was observed.

The implementation of this project was challenging because it took place in a context of human and material resource contingency, due to the COVID-19 pandemic. The results presented are consistent with the improvement in adherence to best practices in screening, prevention, and management of delirium in the PACU, after interprofessional training, highlighting that the adoption of a tool for assessing the delirium is essential for the identification and consequent reduction of the incidence of POD in the PACU. The data obtained support and reinforce the need for investment in evidence-based clinical practice to minimize health risks.

Future audits are required to review and improve compliance with best practices and verify the sustainability of best practices over time.
