# Supplementary material for: Prevention and management of postoperative delirium in adult patients: a best practice implementation project
Source: Rev Esc Enferm USP. 2025 Feb 24;58:e20230426. doi: 10.1590/1980-220X-REEUSP-2023-0426en (PMC11853671; doi:10.1590/1980-220X-REEUSP-2023-0426en)
Supplement: Translation

# Translation: Prevenção e manejo do delirium pós-operatório em pacientes adultos: projeto de implementação de melhores práticas

**DOI:** 10.1590/1980-220X-REEUSP-2023-0426pt

**Authors:** Juliana Rizzo Gnatta, Sarha de Oliveira Gonçalves Paes, Maria Fernanda de Oliveira Faria, Domingos Dias Cicarelli, Renata Veloso Silva Laurino, Lina Hamano, Vilanice Alves de Araújo Püschel, Vanessa de Brito Poveda

**Affiliations:** 1 Universidade de São Paulo, Escola de Enfermagem, Departamento de Enfermagem Médico-Cirúrgica, São Paulo, SP, Brasil.; 2 Universidade de São Paulo, Escola de Enfermagem, São Paulo, SP, Brasil.; 3 Universidade de São Paulo, Hospital Universitário, São Paulo, SP, Brasil.; 4 Centro Brasileiro para o Cuidado à Saúde Baseado em Evidências: Centro de Excelência do JBI, JBI Brasil, São Paulo, SP, Brasil.

**Keywords:** Delírio, Enfermagem em Pós-Anestésico, Complicações Cognitivas Pós-Operatórias, Período de Recuperação da Anestesia, Sala de Recuperação, Pesquisa Interdisciplinar

## Abstract

**Objetivo:**

Descrever o processo de implementação de melhores práticas baseadas em evidências para melhorar a prevenção e o manejo do *Delirium* Pós-Operatório na Sala de Recuperação Pós-Anestésica.

**Método:**

Relato de projeto de implementação de evidências aplicando o JBI fundamentado no processo de auditoria e feedback, com abordagem estruturada para identificação e gerenciamento de barreiras em conformidade com as práticas clínicas recomendadas. Prontuários, sistemas eletrônicos de registros de enfermagem e entrevistas com a equipe foram usados para avaliar as taxas de conformidade.

**Resultados:**

Na auditoria de base, foi encontrada taxa de conformidade nula na maioria dos critérios em relação às melhores evidências. Após essa fase, foram realizados treinamento interprofissional, fornecimento de ferramenta validada para identificar pacientes em risco de *delirium* e alterações no sistema eletrônico de registros de enfermagem. Na primeira auditoria de seguimento houve aumento na taxa de conformidade em quatro dos nove critérios auditados. Quatro critérios alcançaram 100% de conformidade na segunda auditoria de seguimento.

**Conclusão:**

Foram implementadas melhores práticas que contribuíram para melhorar a prevenção e o manejo do *Delirium* Pós-Operatório. Após novo treinamento e reauditoria após um ano, observou-se aumento da adesão às melhores práticas.

## INTRODUÇÃO

O *delirium* é uma condição clínica neuroinflamatória caracterizada por desatenção e nível de consciência instável, apresentando-se como quadro clínico passível de flutuações no decorrer do dia. Ele pode ser classificado em dois tipos: hiperativo e hipoativo. O *delirium* hipoativo é caracterizado por sonolência, com movimentos letárgicos e reduzidos. O *delirium* hiperativo é caracterizado por agitação psicomotora, alucinações visuais e hipervigilância; ambos podem se manifestar imediatamente após o despertar da anestesia na Sala de Recuperação Pós-Anestésica (SRPA)^(1)^.

A incidência de *delirium* varia de acordo com o tipo de cirurgia, acometendo principalmente idosos submetidos a procedimentos cirúrgicos de grande porte, como cirurgias cardíacas, gerais, ortopédicas e vasculares, com acometimento variando entre 10% e 62%^(2,3,4,5)^.

Existem fatores que predispõem os pacientes ao desenvolvimento ou maior risco de *Delirium* Pós-Operatório (DPO). Segundo as diretrizes clínicas propostas pelo *National Institute for Health and Care Excellence* (NICE) para *delirium*, os principais fatores de risco para desenvolvê-lo são idade acima de 65 anos, declínio cognitivo crônico ou demência, fratura de quadril atual, doença grave e condição clínica em deterioração ou em risco de deterioração^(6)^. Além desses, são fatores de risco predisponentes e que aumentam a incidência de DPO na SRPA: a maior duração do período intraoperatório, como o que ocorre em cirurgias de grande porte ou de emergência, comorbidades, anemia pré-operatória, hipotermia, dor pós-operatória e alta glicemia de jejum^(7,8)^.

Compreender a causalidade do DPO é, de fato, importante para oferecer cuidados perioperatórios seguros aos pacientes e prevenir as consequências que dele decorrem, tais como: internação prolongada, perda de independência, redução da função cognitiva e morte^(8,9)^. Assim, a triagem com a identificação e o tratamento precoce do DPO auxiliam na prevenção de complicações futuras. Para tanto, a aplicação de instrumentos validados permite agilidade e precisão na identificação, possibilitando o início do tratamento em tempo oportuno, ainda na SRPA, exigindo trabalho interprofissional para seu correto manejo^(7,10)^.

Para a prevenção de complicações futuras^(7)^, a identificação de indivíduos com risco aumentado para *delirium* possibilita intervenções precoces, melhorando os resultados clínicos do paciente e, consequentemente, o prognóstico^(9)^.

A metodologia de evidências do JBI (*Joanna Briggs Institute*) consiste em oferecer uma abordagem teórica voltada à evidência clínica, utilizando evidências e implementando-as na prática, por meio de auditoria clínica e uso de ferramentas para facilitar e garantir registros de mudanças. É considerada um processo de melhoria da qualidade do atendimento ao paciente e dos resultados obtidos, além de reduzir custos e aumentar a satisfação dos envolvidos no processo de melhoria.

Com base em revisão sistemática e diretrizes clínicas^(7,10,11,12,13)^, o JBI, por meio da publicação de um sumário de evidências^(14)^, faz recomendações classificadas em dois níveis: Grade A, que significa uma recomendação forte, e Grade B, que significa uma recomendação fraca. O JBI recomenda o uso de um teste de triagem cognitiva validado para identificar pacientes em risco, como o *Confusion Assessment Method* (CAM-ICU) ou o *Nursing Delirium Screening Scale* (Nu-DESC) para a detecção de DPO, e o teste deve estar prontamente disponível na SRPA (GRADE B).

Em relação às melhores práticas clínicas, o *Evidence Summary* produzido pelo JBI^(14)^ recomenda que a profundidade da anestesia seja minimizada e monitorada durante a cirurgia (GRADE A). Além disso, o controle multimodal da dor pós-operatória e haloperidol em baixas doses ou neurolépticos atípicos são recomendados quando necessário para o tratamento do DPO (GRADE B). As recomendações descritas foram baseadas em evidências da literatura, de duas revisões sistemáticas^(10,11)^, dois ensaios clínicos randomizados^(12,13)^, um estudo observacional retrospectivo^(15)^ e uma diretriz clínica baseada em evidências^(7)^.

A identificação de pacientes em risco de desenvolver DPO por meio da triagem e detecção contribui para intervenção e manejo precoces, melhoria do cuidado ao paciente crítico, redução do tempo de internação na SRPA, internação e complicações pós-operatórias e melhora do estado do paciente no momento de alta hospitalar.

Anestesiologistas e enfermeiros têm papel importante na prevenção e no manejo do DPO, realizando a avaliação de sinais vitais, hipóxia, hidratação, alimentação (quando indicada), repouso e orientação espaço-temporal, principalmente em casos de cirurgia de emergência^(16)^, intervindo assim para fornecer o melhor cuidado possível com base nas melhores evidências, contribuindo para um melhor manejo do paciente cirúrgico no pós-operatório imediato, que é o foco do presente artigo.

Este projeto de implementação da evidência é interdisciplinar, pois apresenta múltiplas possibilidades de realizações, seja do ponto de vista teórico ou prático, do trabalho em equipe ou da atitude individual do sujeito.

As múltiplas concepções sobre a interdisciplinaridade convergem para a ideia de integralidade, seja ela dos saberes, das aprendizagens, dos sujeitos ou das áreas disciplinares. Faz-se necessário investir no conhecimento sobre a interdisciplinaridade, seja pela atitude individual, coletiva, institucional ou governamental, para implementar as melhores práticas nas instituições de saúde^(17)^.

O objetivo deste estudo foi descrever o processo de implementação de melhores práticas baseadas em evidências para melhorar a prevenção e o manejo do Delirium Pós-Operatório na Sala de Recuperação Pós-Anestésica de um hospital universitário.

## MÉTODO

### Tipo do Estudo

Trata-se de um relato de um projeto de implementação de evidências que utilizou a metodologia de implementação de evidências do JBI, a qual é fundamentada no processo de auditoria e *feedback*, juntamente com uma abordagem estruturada para a identificação e o gerenciamento de barreiras em conformidade com as práticas clínicas recomendadas. Consiste em sete etapas, que são: (1) identificação da área de prática para mudança e do problema juntamente com os *stakeholders*, (2) engajamento de agentes de mudança, (3) avaliação do contexto e prontidão para mudanças (ou seja, análise situacional), (4) revisão da prática (ou seja, auditoria de base) contra critérios de auditoria baseados em evidências, (5) implementação de mudanças na prática aplicando a ferramenta GRiP, (6) reavaliação da prática por meio de uma auditoria de seguimento e (7) consideração da sustentabilidade das mudanças na prática.

Neste modelo, é adotada uma auditoria clínica que propõe uma comparação entre as taxas de conformidade da prática atual com as melhores práticas recomendadas, antes (auditoria de base) e depois (auditoria de seguimento) da implementação da evidência, de forma a avaliar a adesão às melhores práticas. Recomenda-se que sejam realizadas reauditorias periódicas para verificar a sustentabilidade da implementação das evidências ao longo do tempo. O processo é conduzido usando as ferramentas de software do JBI para coleta e análise de dados: *Practical Application of Clinical Evidence System* (PACES)^(18)^ e para identificar barreiras à mudança e propor estratégias para a utilização de evidências na prática: *Getting Research into Practice* (GRiP).

A estrutura de auditoria e *feedback* baseada nas sete etapas do modelo JBI envolveu um processo com três fases: (1) planejamento - identificação da área de prática para mudança e do problema juntamente com os *stakeholders*, engajamento dos agentes de mudança e avaliação do contexto e prontidão para mudanças; (2) avaliação e implementação de linha de base - revisão da prática (auditoria de base) e implementação de mudanças na prática aplicando a ferramenta GRiP; e (3) avaliação de impacto e sustentabilidade - reavaliação da prática por meio de uma auditoria de seguimento e consideração da sustentabilidade das mudanças na prática.

### Local e Amostra

O presente projeto foi desenvolvido na SRPA do Hospital Universitário da Universidade de São Paulo (HU-USP). O hospital está localizado na região metropolitana de São Paulo, Brasil e, durante o período avaliado, contava com 203 leitos. A SRPA contém sete leitos e está inserida no Centro Cirúrgico, onde ocorrem em média 300 cirurgias/mês nas especialidades de ortopedia, cirurgia geral, otorrinolaringologia, ginecologia e oftalmologia. Para compor a amostra do estudo, foram incluídos todos os profissionais que atuavam na unidade no período avaliado. A equipe foi composta por 10 enfermeiros, 39 técnicos de enfermagem e 21 anestesistas, organizados em esquema de rodízio. Não foram estabelecidos critérios de exclusão para a participação.

A equipe de enfermagem da SRPA em cada turno é composta por uma enfermeira, uma técnica de enfermagem por turno (matutino, vespertino e noturno) e um anestesiologista. Os enfermeiros são responsáveis pela aplicação do processo de enfermagem (avaliação, diagnósticos de enfermagem, planejamento, implementação e avaliação) e pela assistência de enfermagem, e os técnicos de enfermagem auxiliam os enfermeiros nas intervenções de complexidade baixa a moderada, como verificação de sinais vitais e cuidados básicos de enfermagem. Os anestesiologistas são responsáveis pela avaliação clínica e pela alta do paciente da SRPA para a unidade de internação, conforme nível de criticidade.

Em 2015, a diretoria da Divisão de Enfermagem Clínica elaborou um protocolo para avaliação e manejo do *delirium* em pacientes clínicos. Apesar de a implementação de melhores práticas em uma unidade de cunho cirúrgico fortalecer a melhora da qualidade da assistência na instituição, a efetivação do referido protocolo não foi estendida a todas as unidades. O protocolo para os pacientes clínicos continua sendo aplicado atualmente e apresenta bons resultados na prática assistencial. Esse foi um dos motivos para se realizar este outro projeto na instituição. Pacientes clínicos ou cirúrgicos beneficiam-se de uma avaliação e manejo sistematizados e adequados sobre *delirium*, diminuindo os eventos adversos e aumentando a segurança, além de contribuir para a qualidade dos processos da instituição.

### Fase 1: Planejamento

A equipe de auditoria envolveu o engajamento das partes interessadas e a identificação dos participantes relevantes da equipe.

A equipe do projeto foi composta por:

– Uma docente com experiência em Enfermagem Perioperatória, líder do projeto, e que já havia atuado como enfermeira assistencial na SRPA da instituição;
– Uma docente com experiência em Enfermagem Perioperatória, que avaliou as estratégias para aumentar o cumprimento dos critérios de auditoria recomendados;
– Três responsáveis clínicos, um chefe da Divisão de Enfermagem Cirúrgica e dois da Divisão de Anestesiologia que disponibilizaram recursos humanos e físicos para as auditorias, desenvolvimento do projeto de implementação e participaram do treinamento da equipe;
– A chefe de enfermagem da Unidade de Centro Cirúrgico, que realizou as alterações necessárias no sistema de prontuário eletrônico para apoiar os registros de cuidados de enfermagem relacionados à triagem e detecção de DPO (escore CAM-ICU) e supervisionou o projeto de implementação;
– Uma residente de enfermagem, que foi responsável por apoiar a formação educacional, acompanhar as dificuldades, dúvidas e barreiras durante a triagem e a avaliação do DPO.

A líder do projeto, a residente de enfermagem e os dois anestesiologistas foram responsáveis pela concepção do projeto, elaboração e condução do programa de treinamento de *delirium*, organização da coleta de dados e elaboração do relatório de implementação.

Os critérios de auditoria baseados em evidências foram derivados do sumário de evidências do JBI sobre as recomendações de melhores práticas em relação à triagem e detecção de *delirium* na SRPA^(14)^. Para definir o nível atual de adesão às melhores práticas, foram considerados todos os nove critérios de auditoria incluídos no JBI-PACES, conforme apresentado no Quadro 1 ^(14)^.

**Quadro 1:** Critérios de auditoria, amostra e método empregado para avaliar a conformidade às melhores práticas de prevenção e manejo do DPO em pacientes adultos na SRPA – Brasil, 2021.

Critério de auditoria*	Amostra	Método usado para medir a conformidade percentual com as melhores práticas
1. A profundidade da sedação durante a anestesia é monitorada subjetiva ou objetivamente.	• Auditoria de base: 10 observações de anestesiologistas durante o procedimento com pacientes com fatores de risco para DPO. • Auditoria de seguimento: 10 observações de anestesiologistas durante o procedimento com pacientes com fatores de risco para DPO.	• Observação direta do anestesiologista durante o procedimento em pacientes com fatores de risco para DPO para verificar se a profundidade da sedação foi monitorada por meio de medida subjetiva ou objetiva. Medida subjetiva: “Sim” − aplicação da escala de sedação; “Não” − sem registro. Medida objetiva: “Sim” − uso de equipamento para guiar a anestesia (ex: *Bispectral Index Monitoring* - BIS); “Não” − não usado.
2. A avaliação de risco pré-operatório para DPO é realizada.	• Auditoria de base: 10 registros de enfermagem de pacientes com fatores de risco para DPO. • Auditoria de seguimento: 10 registros de enfermagem de pacientes com fatores de risco para DPO.	• Revisão dos registros de enfermagem para verificar se havia o diagnóstico de enfermagem “Risco de confusão aguda”. “Sim” − presença do diagnóstico de enfermagem “Risco de confusão aguda”; “Não” − ausência do diagnóstico de enfermagem “Risco de confusão aguda”.
3. A triagem para DPO é realizada o mais precocemente possível na SRPA.	• Auditoria de base: número de pacientes com triagem positiva para DPO em 10 registros em sistemas eletrônicos de enfermagem de pacientes com risco de DPO. • Auditoria de seguimento: número de pacientes com triagem positiva para DPO em 10 registros em sistemas eletrônicos de enfermagem de pacientes com risco de DPO.	• Revisão dos sistemas eletrônicos de registro de enfermagem de pacientes com risco para DPO. “Sim” − se houver pontuação CAM-ICU; “Não” − se não houver pontuação CAM-ICU.
4. As ferramentas *Nursing Delirium Screening Scale* (Nu-DESC) ou Confusion *Assessment Method* (CAM-ICU) são usadas para triagem.	• Auditoria de base: número de pacientes rastreados para DPO usando a ferramenta CAM-ICU em 10 observações de pacientes. • Auditoria de seguimento: número de pacientes rastreados para DPO usando a ferramenta CAM-ICU em 10 observações de pacientes.	• Documentação de enfermagem no prontuário do paciente. “Sim” − presença de escore CAM-ICU registrado no sistema eletrônico de registro de enfermagem; “Não” − ausência do escore CAM-ICU registrado no sistema eletrônico de registro de enfermagem.
5. A ferramenta de triagem é facilmente acessível na SRPA.	• Auditoria de base: presença de instrumento impresso do CAM-ICU disponível na SRPA em 10 oportunidades. • Auditoria de seguimento: presença de instrumento impresso do CAM-ICU disponível na SRPA em 10 oportunidades.	• Presença de cópias do instrumento CAM-ICU na SRPA. “Sim” − Cópia do instrumento CAM-ICU disponível na SRPA para avaliar paciente com fator de risco para DPO; “Não” − Cópia do instrumento CAM-ICU não disponível na SRPA para avaliar paciente com fator de risco para DPO.
6. Os clínicos recebem treinamento para o uso de Nu-DESC e/ou CAM-ICU.	• Auditoria de base: 10 enfermeiros. • Auditoria de seguimento: 10 enfermeiros.	• Entrevista com 10 enfermeiros usando formulário estruturado para verificar se o profissional recebeu treinamento para uso de CAM-ICU no último ano. “Sim” − se o profissional recebeu treinamento; “Não” − se o profissional não recebeu treinamento.
7. Os médicos recebem treinamento sobre os padrões de referência DSM-5 e/ou CID-10.	• Auditoria de base: 10 anestesiologistas. • Auditoria de seguimento: 10 anestesiologistas.	• Entrevista com 10 anestesiologistas utilizando um formulário estruturado. “Sim” − se o profissional recebeu treinamento sobre o DSM-5 e/ou CID-10 no último ano. “Não” − se o profissional não recebeu treinamento sobre o DSM-5 e/ou CID-10 no último ano.
8. O controle multimodal da dor pós-operatória é administrado.	• Auditoria de base: 10 prescrições médicas pós-operatórias acompanhadas da respectiva prescrição anestésica. • Auditoria de seguimento: 10 prescrições médicas pós-operatórias acompanhadas da respectiva prescrição anestésica.	• Revisão de 10 prescrições médicas pós-operatórias para pacientes com risco de DPO que relataram dor durante a internação na SRPA. “Sim” − se o paciente recebeu analgésico opioide combinado com não opioide; “Não” − se o paciente não recebeu analgésico opioide combinado com não opioide.
9. Quando necessário, haloperidol em baixa dosagem ou neurolépticos atípicos em baixa dose são administrados para tratar a DPO.	• Auditoria de base: 10 prescrições médicas pós-operatórias de pacientes com DPO que receberam haloperidol ou neurolépticos atípicos na SRA. • Auditoria de seguimento: 10 prescrições médicas pós-operatórias de pacientes com DPO que receberam haloperidol ou neurolépticos atípicos na SRA.	• Revisão de 10 prescrições médicas pós-operatórias de pacientes com DPO que receberam haloperidol ou neurolépticos atípicos na SRA. “Sim” − se o paciente recebeu haloperidol ou neurolépticos atípicos em baixa dosagem; “Não” − se o paciente não recebeu haloperidol ou neurolépticos atípicos em baixa dosagem.

*Critério de auditoria: baseado no sumário de evidências do JBI das recomendações de melhores práticas^(14)^.

Fonte: os autores.

A auditoria de base incluiu prontuários de 10 pacientes, com idade igual ou superior a 18 anos, que possuíam o diagnóstico de enfermagem “Risco de confusão aguda” e que apresentavam pelo menos um fator de risco para DPO identificado pela residente de enfermagem no momento da admissão do paciente e validado pela enfermeira líder do projeto. Foram incluídos também 10 profissionais de cada categoria (enfermeiros e anestesiologistas) atuantes na SRPA e que atenderam esses pacientes.

### Coleta de Dados

Para a coleta de dados utilizou-se um instrumento impresso criado pelos pesquisadores (Quadro 2) e os dados foram inseridos no PACES. A adesão foi observada pela líder do projeto e pela residente de enfermagem e a coleta de dados realizou-se em novembro de 2021 usando os prontuários de pacientes que apresentavam pelo menos um fator de risco para DPO: idade maior ou igual a 65 anos, comprometimento cognitivo ou diagnóstico de demência, fratura de quadril atual e presença de doença severa^(6)^. A presença da informação foi registrada como “Sim”, para posteriormente medir o percentual de adesão às melhores práticas.

**Quadro 2:** Instrumento de coleta de dados para avaliar a conformidade às melhores práticas de prevenção e manejo do DPO em pacientes adultos na SRPA – Brasil, 2021.

Critérios – Prontuários dos Pacientes	Sim	Não
1. Há registro no Processo de Enfermagem Perioperatória (SAEP) do diagnóstico de Enfermagem “Risco de confusão aguda” no campo para pacientes com risco de *delirium* pós-operatório? (Avaliação de risco pré-operatório para *delirium* – critério 2)		
2. Há registro no prontuário eletrônico (PROCEnf) ou físico da aplicação de um instrumento para rastreio precoce de *delirium* (Ex: Nu-DESC ou CAM) na SRA? (critério 3)		
3. Há registro no prontuário eletrônico (PROCEnf) ou físico da aplicação do instrumento Nu-DESC ou CAM durante a permanência do paciente na SRA? (critério 4)		
4. Há registro na ficha de anestesia ou na prescrição médica do pós-operatório de administração de analgésico não opioide em combinação com opioide? (critério 8)		
5. Há registro na prescrição médica do pós-operatório de haloperidol ou neurolépticos atípicos em baixa dosagem? (critério 9)		
**Critérios – Observação Direta**		
6. O anestesiologista monitorou a profundidade da anestesia por meio de medida subjetiva (ex.: aplicação de escala validada para mensuração da sedação) ou objetiva (ex.: BIS)? (critério 1)		
7. Há disponibilidade para fácil acesso a cópias impressas dos instrumentos Nu-DESC ou CAM ou no sistema eletrônico de registro de dados dos pacientes (PROCEnf)? (critério 5)		
**Critério – Questionamento ao Profissional Anestesiologista**		
8. Nos últimos 24 meses, você realizou algum treinamento sobre os padrões de referência DSM-5 e/ou CID-10? (critério 7)		
**Critério – Questionamento ao Profissional Enfermeiro**		
9. Nos últimos 24 meses, você realizou algum treinamento sobre aplicação dos instrumentos CAM ou Nu-DESC para detecção de *delirium*? (critério 6)		

### Fase 2: Avaliação e Implementação de Linha de Base

Os resultados da auditoria de base foram analisados e compartilhados com a equipe de implementação para identificar barreiras à implementação das melhores práticas.

A ferramenta GRiP do JBI foi aplicada para documentar as barreiras encontradas, as estratégias implementadas e os recursos necessários para superá-las, com o objetivo de melhorar a conformidade aos critérios auditados. As barreiras foram identificadas pelo grupo de profissionais que trabalhavam no local do estudo.

Aproximadamente trinta dias após a conclusão do treinamento, foi realizada uma nova auditoria usando os mesmos critérios da auditoria de base.

### Fase 3: Avaliação de Impacto e Sustentabilidade

A primeira auditoria de seguimento foi realizada em novembro de 2021. Os mesmos critérios de auditoria baseados em evidências, estrutura de amostragem e responsáveis pela coleta de dados (líder do projeto e residente de enfermagem) utilizados na auditoria de base foram aplicados para avaliar a adesão às melhores práticas nas auditorias de seguimento. Após a conclusão da auditoria de seguimento, os resultados foram comparados com a auditoria de base para avaliar as mudanças.

A segunda auditoria de seguimento ocorreu em novembro de 2022. Antes desta auditoria, foi oferecido um novo treinamento para toda a equipe, visto que novos profissionais se inseriram na SRPA após a primeira auditoria de seguimento, porque alguns profissionais já treinados deixaram a unidade. Os profissionais foram treinados em horário oportuno durante o transcorrer do plantão, de modo a não interferir nas atividades laborais. O treinamento foi realizado pela enfermeira residente do projeto e, posteriormente, foram coletados os dados pautados nos mesmos critérios de auditoria baseados em evidências, estrutura de amostragem e da auditoria de base para avaliar a adesão às melhores práticas. Após a conclusão da segunda auditoria de seguimento, os resultados foram analisados e comparados com a auditoria de base.

### Análise dos Dados

Os dados foram incluídos no JBI-PACES e um relatório contendo o percentual de conformidade para cada critério de auditoria de base foi comparado com o percentual das auditorias de seguimento para verificar a mudança da prática clínica em relação à prevenção e ao manejo do DPO.

### Aspectos Éticos

Este projeto foi submetido e aprovado pelo Comitê de Ética em Pesquisa do HU/USP em 21/05/2021 (nº de registro 4.729.079), garantindo a confidencialidade dos dados, bem como a não identificação dos participantes.

Após a autorização das instituições selecionadas para este estudo, pautando-se na Resolução nº 466 de 12 de dezembro de 2012, todos os pacientes e profissionais de saúde foram informados a respeito do objetivo da pesquisa, manifestando sua concordância em participar da investigação, mediante a assinatura do termo de consentimento livre e esclarecido (TCLE), em duas vias, das quais uma foi entregue ao participante e a outra ficou em posse do pesquisador.

## RESULTADOS

### Fase 1: Planejamento

Os resultados da auditoria de base foram analisados usando o percentual de conformidade para cada critério de auditoria antes do programa de treinamento interprofissional. Cada critério de auditoria referente a triagem, avaliação e manejo do *delirium* foi avaliado prospectivamente em 10 prontuários consecutivos de pacientes admitidos na SRPA que apresentavam pelo menos um fator de risco para DPO ou com 10 profissionais de cada categoria que atendiam o paciente.

Não se identificou em nenhum dos prontuários avaliados atendimento aos critérios de auditoria de 1 a 7. A taxa de conformidade nula na maioria dos critérios de auditoria (em 7 dos 9 critérios – 77,8%) da melhor prática era esperada, uma vez que o DPO não estava sendo avaliado por nenhum instrumento válido antes do desenvolvimento do projeto de implementação.

Em relação ao treinamento da equipe (enfermeiros e anestesiologistas) sobre como usar o instrumento recomendado para avaliar o *delirium*, nenhum dos 10 enfermeiros havia sido previamente treinado para aplicar o CAM-ICU e nenhum anestesiologista havia sido treinado nos padrões de referência DSM-5 e/ou CID-10.

### Fase 2: Avaliação e Implementação de Linha de Base

No Quadro 3, são apresentadas as quatro barreiras enfrentadas na implementação das evidências, acompanhadas de estratégias, recursos e resultados alcançados.

**Quadro 3:** Estratégias para colocar a pesquisa na prática – *Getting Research into Practice* (GRiP) – Brasil, 2021.

Barreiras	Estratégia	Recursos	Resultados
1- A maioria dos profissionais de saúde desconhecia os fatores de risco para DPO e como aplicar e interpretar a ferramenta CAM-ICU.	• Desenvolvimento de um treinamento interprofissional formal e acompanhamento dos profissionais no ponto de cuidado para reforçar a aplicação e interpretação do instrumento. • Desenvolvimento de um algoritmo para triar e avaliar o risco de DPO.	• Sessão formal de educação com duração de uma hora envolvendo uma apresentação em PowerPoint, um vídeo educacional, mostrando a prática da aplicação do CAM-ICU. • Aplicação do algoritmo e da ferramenta CAM-ICU, *in loco*, em um paciente na SRPA.	• Os profissionais de saúde conheciam os fatores de risco para *delirium*. Esse desfecho foi medido verificando se os pacientes que apresentavam pelo menos um fator de risco para DPO tinham os diagnósticos de enfermagem “Risco de confusão aguda” ou “Confusão aguda” registrados em seus prontuários.
2- O CAM-ICU não estava disponível na SRPA.	• Fornecimento de cópias impressas do CAM-ICU na SRPA.	• Cópias plastificadas da ferramenta CAM-ICU foram disponibilizadas na SRPA.	• O CAM-ICU impresso estava disponível na unidade. Esse resultado foi medido verificando se a cópia estava disponível na unidade.
3- A triagem e a avaliação do *delirium* não eram registradas nos prontuários.	• Alterações no sistema de registro eletrônico de enfermagem para mostrar a pontuação do CAM-ICU vinculada a diagnósticos de enfermagem apropriados relacionados ao *delirium* (“Risco de confusão aguda” ou “Confusão aguda”).	• Apoio da enfermeira-chefe. • Apoio da informática.	• O número de diagnósticos de enfermagem para “Risco de confusão aguda” ou “Confusão aguda” identificados e casos de DPO aumentou significativamente entre pacientes adultos na SRPA. Esse resultado foi mensurado verificando os registros médicos do paciente e registros de enfermagem eletrônicos.
4- Não há equipamento disponíve1l na instituição para monitorar a profundidade da sedação (BIS*).	• Providenciar a compra de equipamento para monitorar a profundidade da sedação (BIS*).	• Apoio da enfermeira-chefe. • Apoio dos anestesiologistas líderes clínicos.	• Aquisição de equipamento (BIS*). Esse desfecho foi medido verificando se os pacientes que apresentavam pelo menos um fator de risco para DPO foram monitorados pelo BIS durante o procedimento anestésico-cirúrgico.

*BIS = Monitoramento do Índice Bispectral.

A principal barreira identificada pelo grupo foi a ausência de conhecimento dos profissionais sobre os fatores de risco para *delirium*, avaliação pelo CAM-ICU e manejo.

De acordo com a auditoria inicial, nenhum enfermeiro ou anestesiologista havia sido treinado anteriormente em triagem e avaliação de *delirium* com base nos critérios de risco recomendados e na ferramenta CAM-ICU. Por esse motivo, um treinamento interprofissional foi realizado pela líder do projeto, pela residente de enfermagem e pelos dois membros anestesiologistas que compunham a equipe do projeto, apoiados pelos demais membros da equipe de implementação. A estratégia educacional envolveu uma sessão de educação formal de uma hora sobre fatores de risco, triagem, avaliação e manejo do DPO, uma apresentação em vídeo mostrando a prática de aplicação da ferramenta e uma demonstração *in loco* em paciente com “Risco de confusão aguda” admitido na SRPA, usando um algoritmo desenvolvido para o projeto pela pesquisadora e aplicando a ferramenta CAM-ICU.

Todos os enfermeiros de cada turno de trabalho (matutino, vespertino e noturno) foram treinados. No caso dos anestesiologistas, apenas 85,7% concluíram o treinamento porque três profissionais estavam de férias ou afastados de suas atividades laborais durante o processo de implementação.

Outra barreira relevante foi a falta de conhecimento dos enfermeiros sobre como aplicar e interpretar o CAM-ICU. Além disso, a ferramenta CAM-ICU não estava disponível na SRPA. Para superar essa barreira, foi disponibilizado para a unidade o CAM-ICU impresso, ofertado treinamento à equipe e desenvolvido um algoritmo para orientar o profissional a triar, avaliar e classificar o paciente se estava em risco ou se apresentava *delirium*, e, nesse caso, adotar medidas não farmacológicas e comunicar ao anestesiologista, para que indicasse as medidas farmacológicas.

Para superar a barreira quanto à triagem e avaliação do *delirium* em relação ao registro no prontuário, foram feitas alterações no sistema eletrônico de registros do processo de enfermagem. A enfermeira-chefe e o serviço de informática foram responsáveis por apoiar este trabalho.

Após aplicação do instrumento CAM-ICU, os enfermeiros eram responsáveis por identificar os diagnósticos de enfermagem “Risco de confusão aguda” (taxonomia NANDA, código 00173) ou “Confusão aguda” (taxonomia NANDA, código 00128)^(19)^ e registrar no prontuário impresso. Os pacientes CAM-ICU negativos foram diagnosticados como “Risco de confusão aguda” e os CAM-ICU positivos foram diagnosticados como “Confusão aguda”. Em seguida, o enfermeiro preenchia a pontuação do CAM-ICU no sistema de registro eletrônico do processo de enfermagem.

No hospital, não havia equipamento disponível para monitorar a profundidade da sedação. Como a aquisição desse tipo de equipamento contribuiria para a melhoria da prática assistencial e traria mais segurança ao paciente, foi solicitada a aquisição do equipamento *Bispectral Index Monitoring* (BIS).

Dessa forma, 10 enfermeiras (100,0%) e 21 anestesiologistas receberam treinamento (85,7%) sobre identificação de fatores de risco, aplicação prática da ferramenta CAM-ICU, abordagens não farmacológicas e farmacológicas. Aqueles que estavam de férias não participaram da intervenção educativa (três profissionais).

### Fase 3: Avaliação de Impacto e Sustentabilidade

Os resultados da auditoria de seguimento foram analisados usando a porcentagem de conformidade para os nove critérios de auditoria. Cada critério de auditoria foi avaliado em 10 prontuários de pacientes consecutivos admitidos na SRPA com pelo menos um fator de risco para DPO ou por entrevista com 10 enfermeiros e 10 anestesistas. Na Figura 1 são apresentadas as taxas de conformidade da segunda auditoria de seguimento (reauditoria) em comparação à auditoria de base e à primeira auditoria de seguimento.

**Figura 1:** Conformidade (%) da auditoria de base, primeira e segunda auditorias de seguimento com os critérios de melhores práticas para prevenção e manejo do DPO em pacientes adultos na SRPA – Brasil, 2022.

Critérios auditados (legenda):

1- A profundidade da sedação durante a anestesia é monitorada subjetiva ou objetivamente.

2- A avaliação de risco pré-operatório para DPO é realizada.

3- A triagem para DPO é realizada o mais cedo possível na SRPA.

4- As ferramentas Nursing Delirium Screening Scale (Nu-DESC) ou o Confusion Assessment Method (CAM-ICU) são usadas para triagem.

5- A ferramenta de triagem é facilmente acessível na SRPA.

6- Os clínicos recebem treinamento para o uso de Nu-DESC e/ou CAM-ICU.

7- Os médicos recebem treinamento sobre os padrões de referência DSM-5 e/ou CID-10

8- O controle multimodal da dor pós-operatória é administrado.

9- Quando necessário, haloperidol em baixa dosagem ou neurolépticos atípicos em baixa dose são administrados para tratar a DPO.

Em relação à auditoria de base, houve melhora na conformidade em quatro dos nove critérios auditados.

Como o projeto foi realizado no decurso da pandemia de COVID-19, em que os recursos estavam escassos, e em uma instituição pública, onde o processo de compra é realizado por meio de processo licitatório que envolve prazos longos, a aquisição de equipamentos durante o período do projeto foi prejudicada. Assim, a adesão ao critério 1 na auditoria de seguimento foi zero porque o processo de solicitação e aquisição do BIS ainda permanece em tramitação.

Houve aumento em sete dos nove critérios de melhores práticas para prevenção e manejo de DPO na SRPA. A adesão ao critério 1 na segunda auditoria de seguimento também foi zero por não ter sido realizada a compra do BIS para a melhoria da prática clínica.

## DISCUSSÃO

Foi realizada a avaliação de um programa de treinamento interprofissional para melhorar as práticas na prevenção e manejo de DPO em pacientes cirúrgicos adultos na SRPA analisando os critérios para a sua implementação. Na auditoria de base, revelou-se não conformidade com a grande maioria dos critérios auditados conforme as melhores evidências disponíveis, demonstrando que a totalidade da amostra dos pacientes submetidos aos procedimentos cirúrgicos não era sistematicamente triada quanto aos potenciais fatores de risco para desenvolvimento do DPO.

Ainda que o conhecimento sobre os mecanismos subjacentes à fisiopatologia do *delirium* seja limitado, incluindo eventos metabólicos anormais, como desequilíbrio e distúrbios neuro inflamatórios^(19)^, tornam-se irrefutáveis as repercussões negativas no quadro clínico dos pacientes cirúrgicos. Dentre essas repercussões destacam-se: maiores taxas de morbimortalidade, internações hospitalares prolongadas, declínio cognitivo progressivo e aumento dos custos de internação, que resultam em implicações significativas negativas na reabilitação e prognóstico do indivíduo^(19)^.

À medida que os preditores de risco para desenvolvimento do DPO se ampliam, associados à maior complexidade cirúrgica, torna-se essencial a implementação de protocolos e processos de triagem bem estabelecidos envolvendo a equipe interprofissional que atua no período perioperatório^(20)^. A educação continuada e permanente dos profissionais como estratégia facilitadora empreendida pelos serviços de saúde é imprescindível para a detecção dos pacientes com alto risco de evolução para DPO na SRPA, contribuindo assim para limitar sua incidência e oferecer uma assistência de excelência^(20,21,22)^.

Estudos corroboram, sobretudo, a relevância do enfermeiro na triagem e avaliação perioperatória dos grupos com maior risco potencial para desenvolvimento de *delirium*, uma vez que esses profissionais são responsáveis pelo maior quantitativo de tempo destinado à prestação e sistematização dos cuidados diretos aos pacientes^(23,24,25)^. Achados em estudo semelhante sustentam que auditorias de base e acompanhamento, aliadas a um programa de treinamento em *delirium* e mudanças nos registros eletrônicos de enfermagem, aumentaram as taxas de adesão relacionadas à prática baseada em evidências para triagem de pacientes sob risco e avaliação de *delirium* ^(24)^.

Complementando a atuação dos enfermeiros, os membros da equipe de anestesiologia têm a responsabilidade de orientar pacientes e familiares sobre os eventuais fatores de risco, que possam intervir diretamente no gerenciamento e planejamento da alta, em virtude das consequências do DPO^(21)^.

Constatou-se na auditoria de base que, embora os enfermeiros assumam papel importante na prevenção, detecção e manejo do DPO^(23)^, há lacunas no conhecimento desses profissionais nessa temática. Tal fato é corroborado pelos resultados encontrados na auditoria de base, indicando que os enfermeiros, em sua totalidade, não receberam qualquer tipo de treinamento sobre *delirium*. Sendo assim, desenvolveram-se estratégias a fim de prover e instaurar a prática baseada em evidências como preceito indispensável na atuação desses profissionais, aperfeiçoando, consecutivamente, os sistemas operacionais e, sobretudo, a sistematização dos registros da assistência de enfermagem.

A partir da participação dos profissionais nos treinamentos, foi possível definir estratégias de prevenção e manejo do DPO, oportunizando a ampliação de adesão às boas práticas recomendadas. A educação permanente em saúde é um agente modificador de realidades, ao passo que sua implantação nos ambientes institucionais propicia aos colaboradores recursos, motivações, novas experiências práticas e teóricas e, por conseguinte, empoderamento da equipe, criando um ambiente de corresponsabilidade e colaboração mútuas^(25)^.

Diante do exposto, o processo de treinamento dos enfermeiros e médicos anestesiologistas foi idealizado e executado pela equipe do projeto de implementação, que utilizou diferentes estratégias de ensino, tais como: apresentação oral para abordar os principais tópicos sobre *delirium* aplicados ao contexto cirúrgico, como caracterização, sintomatologia, incidência, prevalência e desfechos associados; vídeo explicando a aplicabilidade da ferramenta preditiva de *delirium* − CAM-ICU − e algoritmo para realização das etapas de rastreio, prevenção e manejo do *delirium*. Os profissionais participaram também da aplicação *in loco* do instrumento CAM-ICU e posterior discussão pautada nos questionamentos e dúvidas que surgiam durante a prática. Na aplicação do CAM-ICU logo após o treinamento, os profissionais demoravam cerca de sete a oito minutos. Após a familiarização com a aplicação do instrumento, o tempo dispendido passou a ser cerca de quatro a cinco minutos.

Estudo africano destaca a importância do treinamento *in loco* na prática clínica dos profissionais de saúde, demonstrando aumento significativo na eficácia das estratégias de treinamento para melhorar as práticas dos profissionais^(22)^. Tal estratégia vai ao encontro do resultado obtido no presente projeto, que constatou ampliação nos percentuais de conformidade aos critérios auditados pautados nas melhores práticas após treinamento *in loco*.

A disponibilização contínua de ferramenta confiável aplicada na prática clínica^(7)^ com a finalidade de identificar o *delirium* foi fundamental para respaldar a equipe na avaliação sistemática para prevenção e manejo do DPO, idealizada nos ambientes de admissão e de recuperação pós-anestésica. Tal estratégia contribuiu para facilitar o processo de tomada de decisão pelos profissionais durante o período de recuperação pós-anestésica dos pacientes. Além disso, foi possível identificar que o programa de treinamento desenvolvido favoreceu a triagem e a avaliação de DPO na SRPA no centro cirúrgico, uma vez que houve aumento nas percentagens de conformidade às melhores práticas.

No tocante às ferramentas de registro da assistência de enfermagem, foi importante a atualização do instrumento destinado ao Processo de Enfermagem Perioperatória (SAEP), com inclusão dos diagnósticos de enfermagem “Risco de confusão aguda” e “Confusão aguda” associados à descrição dos fatores predisponentes para o desenvolvimento do *delirium* no pós-operatório. Além disso, foi incluído um campo para descrição do resultado da avaliação do CAM-ICU no sistema operacional eletrônico de enfermagem institucional, no domínio “Cognição”, contribuindo, assim, para documentar a avaliação realizada, facilitar a comunicação entre turnos e unidades, visto que o plantão se configura pelo preceito de continuidade^(25)^.

Em consonância com estudo anterior sobre *delirium*, os resultados obtidos na segunda auditoria de seguimento atingiram índice de conformidade superior a 80%^(26)^, o que reflete êxito do programa de treinamento realizado. No entanto, ainda que o nível de conformidade tenha sido elevado, não foi possível atingir índices absolutos, o que pode ter ocorrido em decorrência da conjuntura situacional de uma organização pública imersa em um contexto pandêmico.

Como limitações, ressalta-se que, na época da implementação do projeto, o mundo passava por uma das maiores pandemias vivenciadas até então, caracterizada pela superlotação dos hospitais. Como consequência, houve contingência de recursos humanos e materiais, sendo necessárias a realocação e a priorização dos gastos. Desse modo, as solicitações de aquisição de materiais para a melhoria da prática clínica foram colocadas em segundo plano, a exemplo do BIS. Uma das recomendações de melhoras práticas para prevenção e manejo do DPO (critério 1) pautava-se na utilização do BIS a fim de que a profundidade da anestesia pudesse ser monitorizada, o que permitiria a titulação mais fidedigna das drogas anestésicas empregadas e poderia reduzir a ocorrência do *delirium* ^(20)^.

Como consequência da pandemia, houve também redução do fluxo cirúrgico. É possível que, ante tamanha demanda assistencial, a equipe tenha priorizado algumas ações em detrimento de outras em face do cenário de múltiplos fatores agravantes vivenciados, o que pode ter influenciado a não adesão total aos critérios de auditoria, especialmente por ser um hospital de ensino e vinculado à melhor universidade da América Latina.

Além disso, a mudança de alguns participantes da equipe e a realização de um novo treinamento após um ano pode comprometer a confiabilidade dos resultados desta pesquisa. Para minimizar esse risco, seria necessária a adoção de treinamento ministrado anualmente para obtenção de melhores resultados na instituição avaliada. Destaca-se que, como houve novo treinamento da equipe interprofissional, não foi possível verificar a manutenção das práticas implementadas durante o período de um ano. Contudo, pode-se evidenciar que um novo treinamento ocasionou maiores taxas de conformidade às melhores práticas (reauditoria) quando comparado com os dados obtidos na primeira auditoria.

Diante dos desfechos obtidos neste estudo, evidenciou-se que a adesão às melhores práticas é possível e factível para identificação de pacientes de alto risco para desenvolvimento de DPO na SRPA e que estratégias de prevenção e manejo interprofissional devem ser implementadas, a fim de prevenir a ocorrência do *delirium* em pacientes cirúrgicos.

## CONCLUSÃO

Foram implementadas melhores práticas que contribuíram para melhorar a prevenção e o manejo do Delirium Pós-Operatório na Sala de Recuperação Pós-Anestésica. Após novo treinamento e reauditoria após um ano, observou-se aumento da adesão às melhores práticas.

A implementação deste projeto foi desafiadora por ter ocorrido em um contexto de contingência de recursos humanos e materiais, em decorrência da pandemia de COVID-19. Os resultados apresentados são condizentes com a melhoria da adesão às melhores práticas em triagem, prevenção e manejo do *delirium* na SRPA, após treinamento interprofissional, ressaltando que a adoção de uma ferramenta para avaliação do *delirium* é essencial para a identificação e a consequente redução da incidência do DPO na SRPA. Os dados obtidos sustentam e reforçam a necessidade do investimento na prática clínica baseada em evidências para minimizar os riscos em saúde.

Auditorias futuras são necessárias para revisar e melhorar a conformidade com as melhores práticas e verificar a sustentabilidade das melhores práticas ao longo do tempo.
